# Distinct Roles of Tensile and Compressive Stresses in Graphitizing and Properties of Carbon Nanofibers

**DOI:** 10.3390/mi12091096

**Published:** 2021-09-11

**Authors:** Yujia Liu, Edmund Lau, Dario Mager, Marc J. Madou, Maziar Ghazinejad

**Affiliations:** 1 Department of Mechanical and Aerospace Engineering, University of California, San Diego, CA 92093, USA; yujial3@uci.edu (Y.L.); e4lau@ucsd.edu (E.L.); 2 Department of Materials Science and Engineering, University of California, Irvine, CA 92697, USA; 3 Institute of Microstructure Technology, Karlsruhe Institute of Technology, 76131 Karlsruhe, Germany; dario.mager@kit.edu; 4 Department of Mechanical and Aerospace Engineering, University of California, Irvine, CA 92697, USA; mmadou@uci.edu

**Keywords:** carbon nanofiber, carbon nanotubes, carbon microstructure, carbon MEMS

## Abstract

It is generally accepted that inducing molecular alignment in a polymer precursor via mechanical stresses influences its graphitization during pyrolysis. However, our understanding of how variations of the imposed mechanics can influence pyrolytic carbon microstructure and functionality is inadequate. Developing such insight is consequential for different aspects of carbon MEMS manufacturing and applicability, as pyrolytic carbons are the main building blocks of MEMS devices. Herein, we study the outcomes of contrasting routes of stress-induced graphitization by providing a comparative analysis of the effects of compressive stress versus standard tensile treatment of PAN-based carbon precursors. The results of different materials characterizations (including scanning electron microscopy, Raman and X-ray photoelectron spectroscopies, as well as high-resolution transmission electron microscopy) reveal that while subjecting precursor molecules to both types of mechanical stresses will induce graphitization in the resulting pyrolytic carbon, this effect is more pronounced in the case of compressive stress. We also evaluated the mechanical behavior of three carbon types, namely compression-induced (CIPC), tension-induced (TIPC), and untreated pyrolytic carbon (PC) by Dynamic Mechanical Analysis (DMA) of carbon samples in their as-synthesized mat format. Using DMA, the elastic modulus, ultimate tensile strength, and ductility of CIPC and TIPC films are determined and compared with untreated pyrolytic carbon. Both stress-induced carbons exhibit enhanced stiffness and strength properties over untreated carbons. The compression-induced films reveal remarkably larger mechanical enhancement with the elastic modulus 26 times higher and tensile strength 2.85 times higher for CIPC compared to untreated pyrolytic carbon. However, these improvements come at the expense of lowered ductility for compression-treated carbon, while tension-treated carbon does not show any loss of ductility. The results provided by this report point to the ways that the carbon MEMS industry can improve and revise the current standard strategies for manufacturing and implementing carbon-based micro-devices.

## 1. Introduction

The effects of molecular alignment of carbon precursors on the microstructural, mechanical, and electrical properties of carbon nanofibers have been studied previously in a number of reports [1,2,3,4,5,6,7,8]. The majority of the reported studies are focused on the more recognized method of air stabilization and carbonization under tensile stress. A standard example of the current trend is the hot-drawing method, where the manufacturing process includes mild heat treatment of stretched polymer fibers prior to or during stabilization. A number of these studies added carbon nanotubes to polymer precursors to boost the alignment of polymer chains via the confinement effect [4,6,8]. While the mechanical treatments have been implemented to different effects in the fabrication of carbon fibers, the mechanism by which they enhance carbon graphitic microstructure and functionality has not been studied conclusively. There are two major questions in this area: (a) how the mechanical treatment of polymer precursors affects the microstructure of the resulting pyrolytic carbons, and (b) if graphitizability is an intrinsic property of certain organic precursors.

Recently, we introduced a mechanism by which electrohydrodynamic forces within the electrospinning process, in conjunction with applied compressive stresses during the stabilization phase, generate graphitic structures in pyrolyzed polyacrylonitrile (PAN) [4]. Generally considered as a “non-graphitizing” precursor, PAN does not undergo a thermodynamically favored molecular reorientation, since its carbonization is devoid of a long fusion step [5]. We hypothesized that this lack of rearrangement of molecules can be overcome by active methods such as an implementation of tensile stress [1,2] or, in our case, the combination of compressive treatment and CNT-originated shear fields [4]. The reported results in these studies were noteworthy in that they offer stress-induced routes to the graphitization of organic precursors and demonstrated the significance of physical conditions implemented during carbon synthesis [1,2,3,4,9].

One of the main unexplored territories within the field of pyrolytic carbons is how the nature of stresses applied during synthesis can alter the microstructure of the resulting carbon. While it is generally accepted that mechanical treatment is advantageous in graphitizing organic precursors, our understanding of how variations of imposed mechanics can influence the carbon microstructure and functionality is inadequate. Answering such questions is crucial, as they may influence different aspects of carbon MEMS manufacturing and applicability. Herein, we study the outcomes of contrasting routes of stress-induced graphitization by offering a comparative analysis on the effects of compressive stress versus standard tensile treatment of PAN-based carbon precursors. In particular, we investigate how reversing the nature of the normal stresses can alter the microstructure, defects, composition, and heterogeneity of the resulting pyrolytic carbon. We also evaluate the mechanical functionality of the two carbon types by comparatively characterizing their fibers’ mechanical behavior in terms of stiffness, strength, and ductility. The results portray interesting differences and provide practical insights into ways that the carbon MEMS industry can improve and revise the current standard strategies for manufacturing and implementing nanocarbon-based devices.

## 2. Materials and Methods

### 2.1. Electrospinning

Polyacrylonitrile (PAN) (Sigma Aldrich, St. Louis, MO, USA) with a molecular weight of 150,000 g/mol, *N*,*N*-dimethylformamide (DMF) (Sigma Aldrich, St. Louis, MO, USA) and multi-walled carbon nanotubes (MWCNT) with a diameter of 110–170 nm and length of 5–9 μm (by Sigma Aldrich, St. Louis, MO, USA, nonfunctionalized and synthesized by CVD growth on Fe substrate) were used to obtain PAN-CNT mats via electrospinning.

The electrospinning solution was obtained by mixing PAN, MWCNT, and DMF with a weight ratio of 8:1:91. To reach a homogeneous solution, the mixture was subjected to ultrasonication for 1 h and then stirred at 60 °C for 24 h to avoid agglomeration of MWCNT.

The PAN-CNT mats with aligned fibers were electrospun by applying a potential of 15 kV between a continuously dispensing syringe (0.25 mL/h) and a rotating, aluminum drum collector (with 7 cm diameter, rotating at 2000 RPM) with a tip-to-axis distance of 15 cm. The mats were removed from the drum after electrospinning for 4 h.

### 2.2. Mechanical Treatment and Stabilization

Prior to stabilization, the as-spun mats were cut into three identical ribbons (approximately 76.2 mm long by 25.4 mm wide). The thickness of ribbons was measured to be 0.229 mm (approximately 0.0090 inch) using a digital micrometer with a ratchet stop from Mitutoyo Corporation, Aurora, IL, USA. A portion of the PAN-CNT ribbons was mechanically compressed for 30 s using an Akiles Pro-Lam Photo Pouch Laminator to further align the carbon molecular chains under 120 °C. Pressure recording film (Fuji Prescale^®^, McMaster Carr, Santa Fe Springs, CA, USA) was used to measure the compression pressure that the carbon fiber mats endured during the lamination compression. The pressure-recording film was cut into the same dimensions as the carbon fiber mat and was subjected to the same compression, upon which the pressure-recording film turned red. Using image processing of the pictures taken from the pressure-recording films and linear regression of pixel intensity vs. color density, the compressive pressure was measured as 835 psi, which is the equivalent of 5.88 MPa. After the compression, the samples were subjected to stabilization oven at 280 °C for 6 h.

For tensile treatment, another portion of as-electrospun carbon nanofiber ribbons were clipped between microscope slides at both ends. Next, a prescribed weight was attached to one end to apply a tensile stress of approximately 52.43 kPa. The set up was hung inside the stabilization oven at 280 °C for 6 h. The remaining portion of as-electrospun nanofiber ribbons were directly subjected to stabilization oven at 280 °C for 6 h without any mechanical treatment as control samples.

### 2.3. Carbonization

The mats were pyrolyzed in an inert, nitrogen gas environment (flow rate of 9000 sccm) inside a Lindberg Blue M tube furnace. To increase graphitization and prevent nanoporosity of the carbon, a two-step pyrolysis process was used. The mats were first heated to 300 °C at a rate of 4.5 °C/min and kept at this temperature for an hour. Then, the temperature was increased to 1000 °C at 2.5 °C/min and held at this value for an hour before cooling down to the ambient temperature.

### 2.4. Characterization

Raman spectroscopy (inVia™ confocal Raman microscope) was performed with a 532 nm laser source to evaluate the graphitic microstructure of pyrolytic carbons. X-ray photoelectron spectroscopy (Kratos AXIS-SUPRA surface analysis instrument) was used to assess the percentage of nitrogen and oxygen groups in the carbon nanofibers. The mechanical characterization was performed by Dynamic Mechanical Analysis (DMA) using a Q800 DMA (TA Instrument, Inc, New Castle, DE, USA) with tensile film clamp. The mats were cut into rectangular ribbons with dimensions recorded. Then, the standard stress and strain tests were obtained with a controlled force ramping rate at 1 N/min until the samples yielded from center.

## 3. Results and Discussion

**Figure 1:**
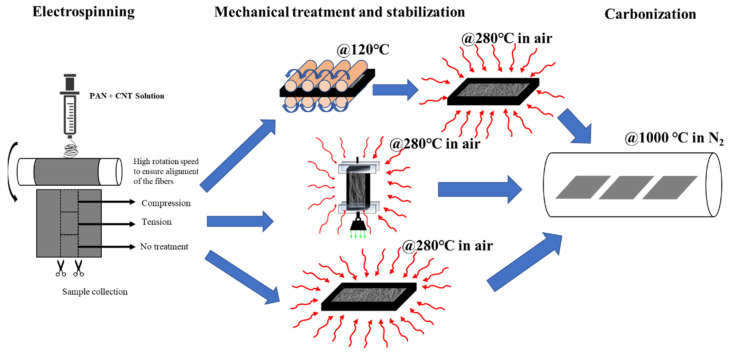
Schematic of manufacturing and stress treatment of PAN/CNT fibers during the stabilization process. After electrospinning PAN/CNT nanofiber film on a spinning drum, samples are produced by cutting identical ribbons from the center of the spun mats to ensure uniform thickness. A portion of the sample is treated with compressive stress, while another portion is subjected to tensile stress and following thermal stabilization in air. The remainder of samples were stabilized without any mechanical treatment and as-spun. Then, all samples were pyrolyzed under nitrogen flow at 1000 °C.

The synthesis route of pyrolytic carbon nanofibers is shown in Figure 1. First, we prepared solutions of PAN precursors that are infused with carbon nanotubes [4,10]. Then, the carbon precursors solution is electrospun into polymer nanofibers mats in a process that is described in detail in our previous reports [4,10,11,12,13]. The electrohydrodynamic forces inherent in the electrospinning process, combined with induced shear fields on the boundary layers of CNTs, unwind the polymer chains and carry out the first stage of molecular alignment in the synthesis process [6,7,14]. Upon electrospinning, some of the polymer nanofiber mats are mechanically rolled and treated under approximately 5.88 MPa compressive stress, while another portion of polymer fibers are treated with a tensile load of 52 kPa. The mechanical stresses augment the alignment of the molecular chains and forge the scaffold of the final carbon structure by confining the polymer chains during the formative cross-linking process in stabilization [1,2,3,4,10,11,12]. Both groups of fiber precursors were stabilized in air at 280 °C and then pyrolyzed under identical conditions at 1000 °C. After implementing fabrication and treatment processes, we analyze the tension-induced pyrolytic carbon nanofibers (TIPC) and compression-induced pyrolytic carbon nanofiber (CIPC) in a series of materials and mechanical characterizations.

### 3.1. Morphology Analysis of Stress-Induced Carbon Nanofibers

**Figure 2:**
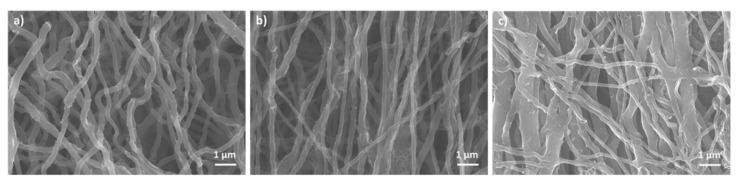
SEM images of carbon samples demonstrate morphologies of PAN-based carbon nanofibers: (**a**) without mechanical treatment, (**b**) tension-induced TIPC, and (**c**) compression-induced CIPC.

Scanning electron microscopy allows us to study the effects of different treatments on the final morphologies of pyrolytic carbon fibers (Figure 2). While all three samples’ nanofibers demonstrate modest alignments in the direction of electrospinning, the tensile treatment of electrospun PAN has resulted in moderately more aligned carbon nanofibers compared to the untreated and compression-treated samples. Using the same micrographs, the average diameter of TIPC fibers measured to be around 241 nm, which is smaller than the average diameter of untreated pyrolytic carbon fibers, which was measured to be 267 nm (please see Figure S1 in the supplementary information).

Conversely, the morphology of CIPC exhibits significant differences from the morphologies of untreated and TIPC samples. From Figure 2c, it appears that the compression treatment of PAN nanofibers under geometric confinement and at 120 °C (which is higher than PAN glass transition temperature) has initiated the cross-linking between the individual electrospun fibers. This phenomenon is highlighted by the larger average diameter of CIPC fibers, measured to be 378 nm, which have a broader size distribution and appear to have “flattened” cross-sections. Accordingly, in some areas of the CIPC fabric, the fibers merged and formed a more continuous layer of carbon film (Figure S2 in Supplementary Information), which is less porous (in terms of macropores) compared to TIPC and untreated carbon samples. The effects of these morphological changes on the mechanical properties of the pyrolytic carbon will be discussed further in the mechanical characterization section.

Another noteworthy aspect of the mechanical treatment is how it affects the thickness of the carbon nanofiber mats, and consequently their porosity. We measured the thickness of all three carbon samples, which was obtained by using ribbons from the same electrospun mat and subjecting them to different mechanical treatment. The thickness of untreated pyrolytic carbon, TIPC, and CIPC ribbons measured as 0.213 mm, 0.196 mm, and 0.036 mm, respectively. The thickness measurements indicate that compression treatment significantly reduces the porosity and creates a closer network of CIPC nanofibers. The SEM micrographs in Figure 2c and Figure S2 corroborate that CIPC has far fewer pores in its tight geometric confinement compared to TIPC and untreated samples. The morphological characteristics and the likelihood of the interaction among the adjacent nanofibers within the pyrolytic carbon framework is an additional factor that, along with graphitization level, contributes to the mechanical response of stress-induced carbon samples. We will discuss this aspect further in the Section 3.5.

### 3.2. Structural Analysis of CIPC and TIPC by Raman Spectroscopy

**Figure 3:**
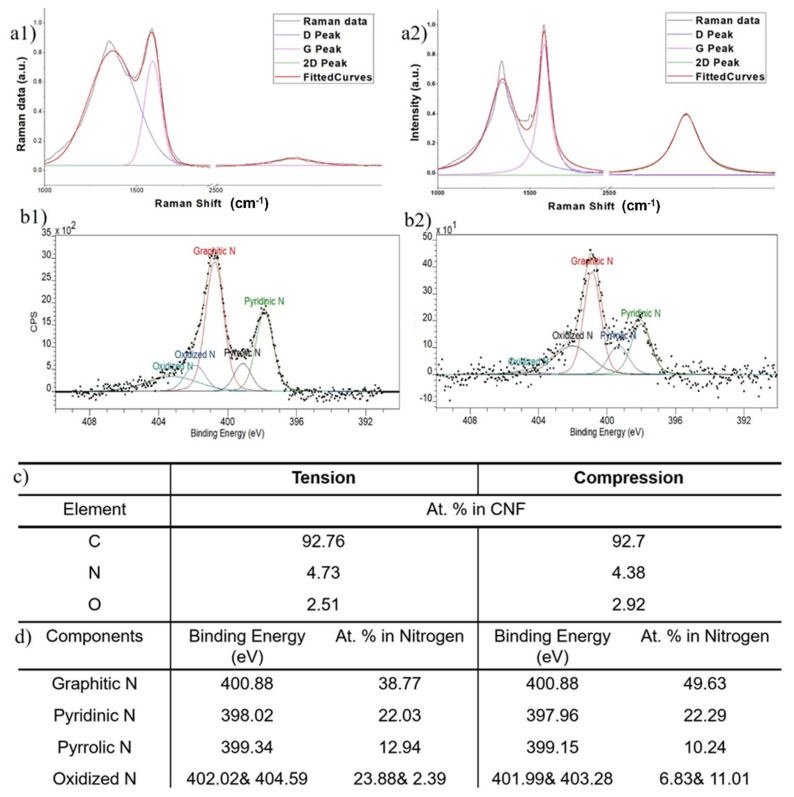
(**a**) Raman spectroscopy of (**a1**) tension-induced (TIPC) and (**a2**) compression-induced (CIPC) pyrolytic carbons (*λ_excitation_* = 532 nm). (**b**) XPS spectra of the N 1s peaks of (**b1**) TIPC and (**b2**) CIPC. (**c**) Table of elemental composition of the TIPC and CIPC as determined by analysis of XPS spectrum. (**d**) Distribution of graphitic, pyridinic, and pyrrolic nitrogen as the main heteroatoms in TIPC and CIPC, as determined by analysis of XPS spectrum.

We open our analysis with a standard Raman spectroscopy of pyrolytic carbons. Subsequent to a considerable number of theoretical and experimental studies dedicated to the Raman spectroscopy of carbon nanomaterials in the past few decades [8,15], this method has been established as a standard tool to gain swift, reliable insights into the structures and defects of carbon specimens. Herein, we used Raman to mainly evaluate the graphitic quality of our stress-activated carbons (Figure 3a). The main metrics of interest for our analysis are the values associated with D and G Raman peaks. The intensity and shape of G peaks is associated with the stretching motion of in-plane c–c bonds with sp2 hybridization. Thus, it represents the graphitic building blocks and crystallinity. The D is a disorder-originated peak and is associated with defects and divergence from the graphitic carbon structure. Figure 3a displays the Raman spectra of TIPC (1-a1) and CIPC (1-a2).

Our first comparison between Raman spectra gives a clear edge to CIPC in terms of graphitic quality. Here, while CIPC demonstrates *I_D_*/*I_G_* = 0.71, TIPC registers a value of 1.13 for this ratio. The Raman results of tension-treated carbon fibers are consistent with similar studies using similar fabrication procedures [9,16]. The inferior graphitic quality of TIPC fibers could be caused by the lower tension threshold of the carbon fibers, which leads to microtears and the disruption of PAN fibers prior to pyrolysis. The presence of partial microtears—inevitable due to the nature of applied loading—in PAN yarns would locally relieve a number of nanofibers from tension (as they are dangling within PAN yarn) and essentially render the mechanical alignment of their molecular chains ineffective. Then, the subsequent stabilization will result in cross-linking among randomly curled precursor chains, yielding amorphous carbon regions.

The comparison between the pick location of CIPC and TIPC does not display any particular pattern. This is expected, as the high-temperature, lengthy pyrolysis step removes the residual mechanical stresses in the final carbons. It should be noted that it is inherently more difficult to control the exertion of tensile forces and produce uniform nanofiber fabrics. A significant number of materials (particularly brittle ones) tend to have lower tolerance for tensile loading compared to compressive loading. This phenomenon, which is due to the presence of discontinuities and micro-flaws, makes the tensile treatment of carbon precursors more challenging, as any effort to align their molecular chains via tensile stresses is capped by their lower limit of tensile strength. A method used in the carbon fiber industry to address this issue is the application of hot drawing, where polymer fibers are woven into robust yarns and are collectively stretched. The robustness of yarn and the intertwined nature of fibers will distribute the tensile stress more uniformly and mitigate the number of microtears. However, the Raman spectroscopy results of the carbon fibers manufactured by this method remain very similar to those of TIPCS reported here, pointing to the inherently different effects of tension and compression treatment [1,9].

### 3.3. Chemical Composition Analysis of CIPC and TIPC

In the next part of our analysis, we investigate how the nature of mechanical loading applied to PAN precursors (compressive or tensile) can influence the chemical bonds and amount of heterogeneous atoms in the resulting pyrolytic carbon fibers. In a previous study, we reported that a sizable amount of graphitic and pyridinic nitrogen atoms is preserved in the stress-activated carbons. We attributed the presence of these atoms to the low temperature of our pyrolysis step, typically carried out at 900–1000 °C, which is far lower than current industry standards (typically 1500–3000 °C). This is advantageous from different aspects; first, fabricating graphitic carbon at a lower pyrolysis temperature is remarkably more cost-effective; second, the presence of functional heterogeneities (such as pyridinic nitrogen) will positively contribute to the electrochemical performance of carbon nanofibers [12,17,18,19].

To compare the chemical composition of TIPC and CIPC, we performed X-ray Photoelectron Spectroscopy (XPS) and curve fitted the collected spectra to deconvolute the nitrogen peak, N 1s (Figure 3b–d). The comparison of the XPS data drawn from both carbon types reveals that the total amount of the nitrogen—the main heterogeneous atom in pyrolytic carbon—maintains a similar fraction of approximately 4.7% in both carbons. However, the type of nitrogen exhibits an interesting variation from compression to tension. While CIPC demonstrates high contents of graphitic nitrogen (49.63%) and a small amount of pyrrolic nitrogen (10.24%), TIPC contains noticeably lower graphitic nitrogen (38.77%) and higher pyrrolic nitrogen (12.94%) concentrations. The fraction of pyridinic nitrogen remains similar (22.29% for CIPC and 22.03% for TIPC) in both carbons. TIPC also possesses a higher percentage of oxidized nitrogen (23.88% and 2.39%). The observations drawn from XPS analysis will be specifically meaningful in the context of carbon MEMS and microelectrodes, as graphitic and pyridinic nitrogen are reported to contribute positively to carbon’s electrochemical performance [10,11,20,21].

Overall, both carbon fibers show a similar breakdown of total carbon, nitrogen, and oxygen (Figure 3c—top table). However, they exhibit a noticeable disparity in the amounts of graphitic, pyridinic, and pyrrolic nitrogen atoms embedded in their structures.

### 3.4. Transmission Electron Microscopy and Microstructure

**Figure 4:**
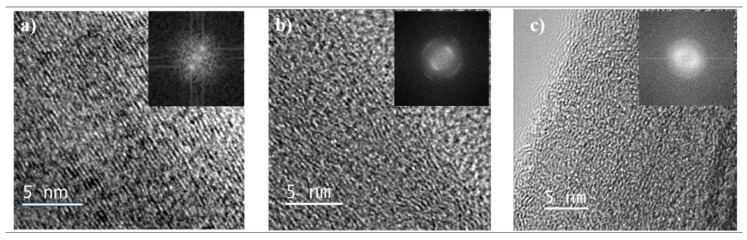
High-resolution TEM images of PAN-based carbons: (**a**) compression-induced CIPC, (**b**) tension-induced TIPC, and (**c**) without mechanical treatment. Micrograph (**c**) is provided for comparing the microstructure of mechanically activated carbons (**a**,**b**) with untreated (no mechanical activation) PAN-based pyrolytic carbon (**c**). The inset in the upper right corner of each micrograph displays the Fast Fourier Transform (FFT using a Gatan Digital micrograph) of the microstructure of each PAN-based carbon.

To compare the microstructure of CIPC and TIPC, we employed transmission electron microscopy (TEM) imaging. While limited in its field of view, this high-resolution visual technique can offer nano-scale insights into the microstructure of the two carbons. Figure 4 shows the high-resolution TEM images of CIPC (Figure 4a) and TIPC (Figure 4b). Figure 4c is obtained from PAN/CNT-based pyrolytic carbon synthesized without mechanical treatment, but otherwise under the same synthesis parameters as CIPC (Figure 4a) and TIPC (Figure 4b). Figure 4c provides us with a ground for comparison to see the general impact of mechanical activation (compressive and tensile) on the pyrolytic carbon microstructure.

Overall, both mechanically activated carbons (Figure 4a,b) show an enhanced level of alignment of carbon planes in comparison with mechanically untreated carbon and amorphous carbon microstructures [4]. Although both stress-induced carbons show similar microstructures, the CIPC carbon fringes appear to be slightly more aligned than those of TIPC. Moreover, the TIPC structure appears to be coarser with more broken carbon fringes, more undulations, and less ordering. This is consistent with the results of RAMAN spectroscopy analysis, which indicates a higher graphitization degree in CIPC fibers. On the other hand, the untreated sample (Figure 4c) exhibits a disordered and haphazardly curled microstructure, which is in keeping with the accepted classification of PAN as a non-graphitizable polymer. Furthermore, the Fast Fourier Transform (FFT) of the Figure 4c micrograph reveals symmetric rings, which is indicative of an amorphous carbon microstructure.

The carbon fringe separation distance (d spacing), derived from processing of the TEM images, corroborates the visual observation of microstructures of the two mechanically induced carbons. Here, TIPC shows an average d spacing of 3.65 Å, while CIPC’s fringe separation is 3.55 Å. The difference implies a closer stacking among CIPC carbon planes, which can be attributed to the nature of compression treatment of carbon precursors. As a reference, the d spacing for most of the carbon nanotubes and highly organized pyrolytic graphite is usually under 3.4 Å [4,22,23]. An interesting element of the microstructure in carbon fibers is the trade-off between ordered graphitic structures and the amount of carbon edge planes. While the quantity of carbon edges is a main contributor to carbon MEMS/electrode’s performance, too much fragmentation of the carbon lattice could disrupt the graphitic network necessary for efficient electron mobility in carbon electrodes.

### 3.5. Mechanical Characterization of CIPC and TIPC

One of the most tangible synthesis–structure–property relationships in carbon can be studied by mechanical characterization of pyrolytic carbons manufactured through different routes. Mechanical characterization can be done on different scales, ranging from nano-scale/single fiber testing to macro-scale evaluation of carbon fiber mats [2,16,24,25]. For this study, we decided to obtain the mechanical properties of the stress-induced carbons in their as-pyrolyzed mat format. Assessing the collective properties of carbon nanofibers in functional geometries, such as carbon mats incorporated in electrodes, allows us to predict the mechanical behavior of these materials when used in sensing and storage devices [26,27,28,29]. While focusing on a single nanofiber has certain scientific advantages, the mechanical properties obtained from a single fiber analysis cannot be easily translated to larger, functional dimensions. Conversely, measuring the mechanical behavior of carbon fabrics in device-relevant dimensions is directly applicable. It is also important to note that the results of mechanical characterization of the larger carbon films may not be directly compared to single fiber characterization due to additional factors in bulk dimensions, such as the porosity of the fiber mat, nanofiber dispersity, and fibers’ orientations. [16,24,25].

Dynamic Mechanical Analysis (DMA) allowed us to accurately characterize the mechanical behavior of the delicate carbon mats. The main objective of mechanical characterization for this study is to obtain reliable stress–strain curves for the samples. As the focus is to assess the behavior of carbon samples for device integration, we used the engineering definition of stress $=\frac{F}{A}$, where *A* is the initial cross-sectional area of the strip and *F* is the forces applied on the carbon fabric strips. Using a tensile film clamp, the standard stress and strain tests were conducted using a controlled force ramping rate at 1 N/min until the samples yielded from the center.

**Figure 5:**
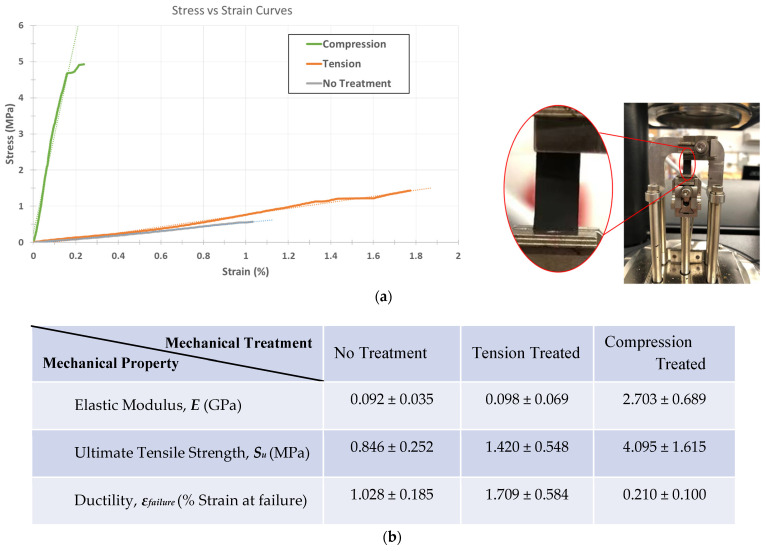

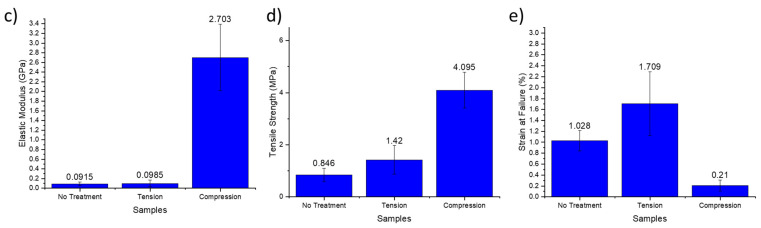
(**a**) Right: Representative stress–strain diagrams of all three carbon samples; Left: Photo of Dynamic Mechanical Analyzer with the pyrolytic carbon nanofiber mat attached to tensile test fixtures, (**b**) table of mechanical properties of the different types of carbons, and (**c**–**e**) bar graphs illustrating the differences in elastic modulus, ultimate tensile strength, and ductility in CIPC, TIPC, and no-treatment carbon samples.

First, we focus on the elastic response of each sample as the most important aspect of mechanical behaviors. As shown in Figure 5, the compression-induced pyrolyzed carbon demonstrates a remarkable increase in elastic modulus (*E*_CIPC_ = 2.703 GPa) with a 29-fold rise over the Young’s modulus of the control untreated pyrolytic carbon (*E*_untreated_ = 92 MPa).

Tension treatment also slightly enhances the stiffness of carbon fiber to *E*_TIPC_ = 98 MPa, a 6.5% increase, which is far less than the effect of compression treatment. It is important to note that the over-tensioning of carbon precursors in our experiments often results in compromised/partially torn carbon fiber mats, where the defects are not visually detectable without microscopy. In these cases, over-tensioned carbons will be even weaker than untreated pyrolytic carbons due to the structural damages. Therefore, it is necessary to modulate the tension treatment to an acceptable limit to see its strengthening effect. The elastic behavior results obtained in the current study agree with the previously reported correlation between carbon’s stiffness and its graphitic quality [30,31,32]. Another parallel between carbons’ composition–microstructure and mechanical behavior is the higher concentration of graphitic C–N bonds in CIPC compared to TIPC, which is a likely contributor to a more graphitic microstructure, and consequently, higher stiffness in compression-induced carbon fibers [30,31,32].

Another notable observation is the change in ductility of the carbon fiber fabrics upon mechanical treatment. Using strain at failure as a measure of ductility, DMA shows that while compression treatment drastically boosts the elastic response of pyrolytic carbon, it lowers its strain at failure to *ϵ_f_* = 0.21%, denoting a significant drop in carbon mats’ ductility. Interestingly, this pattern is not observed in the tension-treated carbon mats, as they exhibit an enhanced ductility with (*ϵ_f_* = 1.71%) compared to that of the control, untreated mat with (*ϵ_f_* = 1.03%). The observed ductility trend is mostly for thin film carbon fabrics used as electrodes for sensing and electrochemical application; if the pyrolytic mats are woven into carbon yarns, the result may vary [30,31,32,33,34,35,36].

The ultimate strength, S_U_, of carbon fabrics is another mechanical property that is crucial to carbon device integration. In many applications, the carbon nanofiber fabrics should withstand different loading scenarios while functioning as electrodes and sensing probes. This mechanical tolerance for loads is usually constrained by the sample’s ultimate strength (Figure 5). Both CIPC and TIPC show a promising enhancement in their ultimate strength, with TIPC registering 68% enhancement (*S_U_*_,TIPC_ = 1.420 MPa) and CIPC demonstrating a 484% increase (*S_U_*_,TIPC_ = 4.095 MPa) in their ultimate strength values. Combined with their elastic behavior, the mechanical characterization suggests that compression treatment enhances the stiffness and strength of pyrolytic carbon mats notably more than tension treatment at the cost of making them more brittle.

It is beneficial to explore the effects of the morphologies of different carbon nanofibers on their mechanical properties. From the SEM micrographs, tensile treatment—performed along the electrospinning direction—has enhanced the alignment of carbon nanofibers in TIPC mats. This augmented alignment is likely another factor that contributes to the increase in TIPC’s elasticity and strength, which are measured along the electrospinning/treatment direction. Conversely, in the case of CIPC mats, the compression treatment of spun fibers has visibly resulted in the inter-fiber cross-linking and formation of semi-continuous carbon layers during the pyrolysis (Figure S2). The enhanced continuity and inter-connection of nanofibers results in a more rigid 2D, less porous carbon framework, which exhibits a significant surge in stiffness and strength. However, such continuity can lead to reduced ductility in CIPC, as a small tear in the carbon film can propagate and result in failure at smaller strains compared to more porous TIPC and untreated mats.

The mechanical, structural, and morphological characterizations in the current study suggest that the morphology and alignment of carbon nanofibers, along with their graphitization level (an established factor on the mechanical properties of carbon) are among the main contributors to the mechanical properties of stress-induced pyrolytic carbons. While this report provides useful insights on the mechanical behavior of carbon nanofibers, the influence of fiber alignment and testing direction on the mechanical response of carbon fibers requires further investigation, which the authors plan to pursue in a future study.

The mechanical behavior has numerous characteristics, including but not limited to fracture toughness, rigidity, resilience, etc. The complete mechanical characterization of the carbon fibers merits a dedicated study that explores the dimension of the pyrolytic carbon samples (such as micro-/single fiber, meso-, and macro-scale) as well as the scale of characterization and the effects of nanofibers’ orientation and alignment. The current study provided an overview of the methodology and main mechanical characteristics of mechanically induced pyrolytic carbons in the geometries relevant to devices integration. This report will be followed up with further studies of the mechanics of stress-induced pyrolytic carbons in our future works.

## 4. Conclusions

Although compression and tension are both considered normal stresses in continuum mechanics, they can cause different physical behavior in material systems that are subjected to them. The different consequences of these two mechanical loadings are particularly noticeable in anisotropic materials, such as nanofibrous PAN films fabricated by electrospinning. Ninety percent of the commercial carbon fibers are manufactured by pyrolyzing spun PAN fibers, which are regularly subjected to tensile treatment to enhance the properties of the carbons that they produce. Accordingly, it is important to comparatively study the outcome of tensile and compressive stresses on PAN-based carbon nanofibers that are routinely used in carbon MEMS and affect the functionality of these micro-devices.

We fabricate tension-induced (TIPC) and compression-induced (CIPC) graphitic carbon by treating electrospun PAN nanofibers (as carbon precursors) with tensile and compressive stresses, respectively. The stress treatments are applied prior to and during the cross-linking of polymer precursors in the stabilization step. After pyrolysis, we characterize both carbons to comparatively analyze the effects of the two types of stresses. Raman spectroscopy and HRTEM characterizations reveal that while subjecting precursor molecules to mechanical stresses induces graphitization in all pyrolytic carbons, this effect is more pronounced in the case of compressive stresses. This result is particularly useful, as compressive stresses can be applied more accurately and over well-defined patterns with strategies such as nanoimprinting. The microstructures of both carbons are innately rich in carbon edge planes, which is an advantageous feature for electrochemical performance and MEMS device integration. Moreover, XPS analysis of samples indicated that while both carbons are inherently rich in nitrogen heteroatoms, CIPC contains a higher concentration of graphitic and pyridinic nitrogen. Finally, Dynamic Mechanical Analysis (DMA) allowed us to investigate the mechanical behavior of CIPC and TIPC in their mat format. Both as-synthesized carbons exhibit enhanced mechanical performances in terms of elastic modulus and ultimate tensile strength, with CIPC registering far exceeding stiffness and strength compared to tension-treated and untreated carbon nanofiber films. However, the strain-to-failure values derived from DMA reveal that compression treatment notably reduces carbon fibers ductility in the process, while tension treatment enhances ductility.

Our findings demonstrate an interesting facet of pyrolytic carbon synthesis, where the compression of polymer precursor results in better graphitization and mechanical properties. The benefits of compressive treatment are even more noteworthy when one considers that compressive stress can be applied more selectively and uniformly across polymer precursors fabrics. Additionally, the availability of high-resolution methods for exerting compressive stress, such as nanoimprinting, points to an inherent advantage of the compression–activation for microfabricating high-performance carbon-based devices.

## Supplementary Materials

The following are available online at https://www.mdpi.com/article/10.3390/mi12091096/s1. Figure S1: Measuring diameters of different carbon nanofibers using the SEM images of PAN-based carbon samples: (a) without mechanical treatment, (b) tension-induced TIPC, and (c) compression-induced CIPC. The average diameter of each type of carbon nanofibers are obtained by averaging the measurements in each SEM micrograph. Figure S2: Compression treatment of PAN nanofibers at 120 OC, has initiated the crosslinking between the individual electrospun fibers. In many areas across CIPC fabric, the nanofibers merged and formed a more continuous layer of carbon with a lower porosity compared to untreated and TIPC samples. Additionally, CIPC nanofibers have larger average diameters and appear to have “flattened” cross sections. Table S1: Average nanofiber diameters for untreated PC, TIPC, and CIPC with standard deviation, obtained from the SEM micrographs in Figure S1.
