# Supplementary material for: Distinct Roles of Tensile and Compressive Stresses in Graphitizing and Properties of Carbon Nanofibers
Source: Micromachines (Basel). 2021 Sep 11;12(9):1096. doi: 10.3390/mi12091096 (PMC8466282; doi:10.3390/mi12091096)
Supplement: Supplementary file 1 [file micromachines-12-01096-s001.zip › micromachines-1346757-supplementary.pdf]

Supplementary Information

Distinct roles of tensile and compressive stresses in graphitizing pyrolytic carbon nanofibers

Manuscript Number: ***micromachines-1346757***

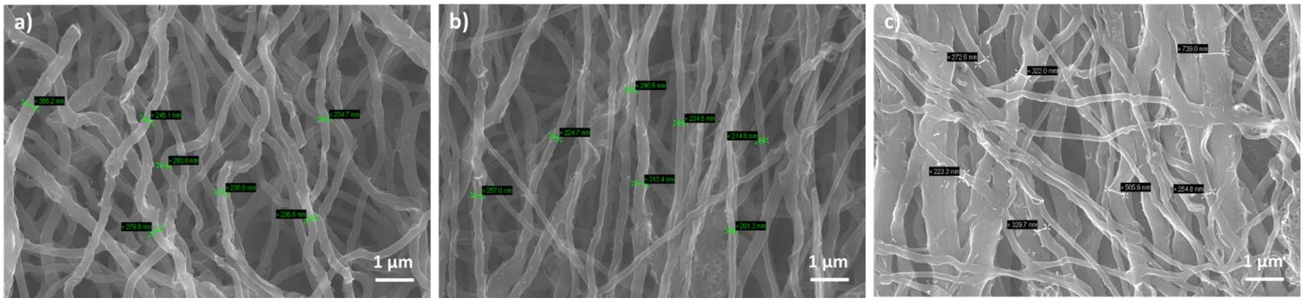

Figure S1. Measuring diameters of different carbon nanofibers using the SEM images of PAN-based carbon samples: (a) without mechanical treatment, (b) tension-induced TIPC, and (c) compression-induced CIPC. The average diameter of each type of carbon nanofibers are obtained by averaging the measurements in each SEM micrograph.

Sample Treatment	Average Fiber Diameter (nm)	Standard Deviation (nm)
**No Treatment, Fig S1-a**	**267.0**	**44.1**
**TIPC, Fig S1-b**	**241.8**	**36.7**
**CIPC, Fig S1-c**	**378.2**	**183.7**

Table S1. Average nanofiber diameters for untreated PC, TIPC, and CIPC with standard deviation, obtained from the SEM micrographs in Figure S1.

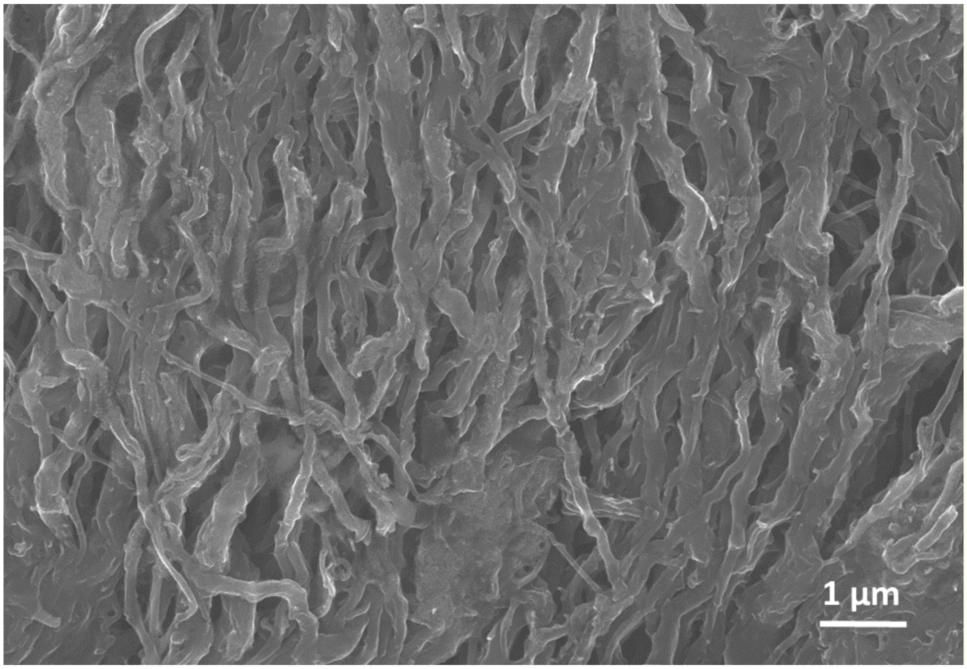

Figure S2. Compression treatment of PAN nanofibers at 120 ^{O}C, has initiated the crosslinking between the individual electrospun fibers. In many areas across CIPC fabric, the nanofibers merged and formed a more continuous layer of carbon with a lower porosity compared to untreated and TIPC samples. Additionally, CIPC nanofibers have larger average diameters and appear to have "flattened" cross sections.
